# Cytokine Profiling in Cutaneous Melanoma: The Emerging Role of Interleukins in Prognostic Stratification with an Up-to-Date Overview of Published Data

**DOI:** 10.3390/jpm16020120

**Published:** 2026-02-15

**Authors:** Paola Negovetić, Klara Gaćina, Nika Franceschi, Marija Buljan

**Affiliations:** 1 Department of Dermatology and Venereology, University Hospital Centre Sestre Milosrdnice, 10000 Zagreb, Croatia; 2 School of Dental Medicine, University of Zagreb, 10000 Zagreb, Croatia

**Keywords:** melanoma, dermatology, cytokine, interleukin

## Abstract

**Background:** Cutaneous melanoma is an aggressive malignancy driven by complex interactions between tumor cells and the host immune system. Tumor progression is shaped not only by intrinsic tumor characteristics but also by immune-mediated processes within the tumor microenvironment. Cytokines, particularly interleukins, are key regulators of inflammation, immune cell recruitment, and tumor behavior. Cytokine profiling provides an integrated assessment of soluble immune mediators from tumor and stromal cells, reflecting both local and systemic immune responses. **Methods:** This narrative review summarizes and synthesizes the current literature addressing the biological and clinical relevance of selected interleukins, including IL-6, IL-8, IL-10, IL-2, IL-17, and IL-18, in cutaneous melanoma. Published data were evaluated with a focus on their immunomodulatory functions and potential implications for prognostic assessment. **Results:** Interleukins demonstrated distinct and context-dependent prognostic and predictive relevance in cutaneous melanoma. Elevated IL-2 levels correlated with sentinel lymph node positivity, supporting its prognostic value in early disease. Increased circulating IL-6 and IL-8 were consistently associated with tumor burden, advanced disease, and reduced survival. IL-10 expression reflected tumor progression and immune modulation. IL-17 signatures predicted response to combined immune checkpoint inhibition, particularly in BRAFV600-mutant melanoma. IL-18 exhibited dual roles, associating with both immune activation and favorable outcomes depending on tumor context. **Conclusions:** Interleukin profiling offers a biologically relevant framework for understanding immune regulation in cutaneous melanoma. Integrating interleukin signatures into prognostic models may support more refined risk stratification and advance the implementation of personalized medicine approaches in melanoma management.

## 1. Introduction

Cutaneous melanoma represents a highly aggressive skin malignancy characterized by complex interactions between tumor cells and the host immune system. Melanoma progression is regulated not only by intrinsic tumor characteristics but also by dynamic interactions within the tumor microenvironment, where immune mediators play a central regulatory role [1]. In this context, cytokines, particularly interleukins, have emerged as critical modulators of melanoma biology, influencing tumor progression, immune infiltration, and therapeutic response. Cytokine profiling refers to the comprehensive assessment of soluble factors released by both malignant and stromal cells, enabling characterization of the tumor microenvironment and systemic immune response [2].

Interleukins such as IL-2, IL-6, IL-8, IL-10, IL-17 and IL-18 have been repeatedly implicated in melanoma pathogenesis and prognosis [3,4,5]. In melanoma, dysregulated cytokine networks influence tumor development, angiogenesis, immune evasion, and metastatic spread. Among these mediators, interleukins establish a highly diverse family with pleiotropic effects on both innate and adaptive immunity. Their context-dependent activity reflects the balance between tumor-promoting inflammation and effective anti-tumor immune surveillance, making them particularly important to melanoma biology [6].

The evolution of multiplex immunoassays and high-throughput profiling techniques has enhanced cytokine detection capabilities, enabling more comprehensive stratification frameworks. Emerging evidence indicates that interleukin profiles may not only reflect baseline tumor biology but also predict responses to immunotherapies and targeted therapies. Continued integration of cytokine profiling into clinical and translational melanoma research holds promise for improving prognostic accuracy and guiding personalized management strategies [7].

Prognostic biomarkers are defined as markers that provide information on disease outcome independent of therapy, whereas predictive biomarkers indicate the likelihood of response or toxicity to a specific treatment. Throughout this study, interleukins are described as prognostic when associated with survival or disease progression across treatment modalities, and as predictive only when linked to differential benefit or toxicity from a defined therapeutic intervention.

The aim of this narrative review is to synthesize the current evidence on the role of interleukins in the biology, prognosis, and therapeutic response of cutaneous melanoma. The literature was identified by a targeted search of PubMed, Scopus and Web of Science databases, covering studies published up to 2025. Search terms included combinations of “cutaneous melanoma,” “interleukins,” “cytokines,” “tumor microenvironment,” “biomarkers,” and “immunotherapy.” Preference is given to original research articles, translational studies, and clinically relevant cohort studies published in peer-reviewed journals, with an emphasis on human melanoma, clinically annotated cohorts, and experimental work that provides mechanistic or translational insight; non-melanoma studies, isolated case reports, and publications lacking biological or clinical relevance were not prioritized. No formal systematic review methodology or risk-of-bias assessment was applied, and study selection was guided by relevance and biological context. Although no formal systematic review methodology was applied, the goal of the study selection was to provide a comprehensive and biologically contextualized overview of interleukin-mediated pathways in melanoma.

## 2. Interleukin 2

Interleukin-2 (IL-2) is a pleiotropic cytokine identified as a T-cell growth factor that plays a central role in regulating immune responses [8]. It consists of a protein that signals through the heterotrimeric IL-2 receptor (IL-2R) complex, and IL-2 orchestrates the proliferation, survival, and differentiation of various lymphocyte subsets, including T-cells and natural killer (NK) cells. The balance of IL-2 signaling critically shapes the tumor immune microenvironment, where it can enhance antitumor immunity but also contribute to immunoregulatory networks that suppress effective responses [9,10].

In physiological contexts, IL-2 maintains immune homeostasis by supporting the expansion of effector T-cells and maintaining regulatory T cell populations that limit autoimmunity. Within malignancies such as cutaneous melanoma, the pleiotropic nature of IL-2 has particularly significant implications [11]. On the one hand, IL-2 can enhance antitumor immunity by supporting the expansion and cytotoxic activity of tumor-reactive T cells and NK cells. On the other hand, preferential expression of high-affinity IL-2 receptors on regulatory T cells can lead to their selective expansion, contributing to immunosuppressive networks within the tumor microenvironment. This dynamic interplay between immune activation and regulation highlights the complexity of IL-2-mediated signaling in melanoma and highlights its central role in modulating the efficacy of antitumor immune responses [6,11,12].

Recombinant human IL-2 became among the first immunotherapeutic agent approved for the treatment of advanced melanoma, based on clinical evidence demonstrating durable complete responses in a subset of patients with metastatic disease [13]. The clinical effects of IL-2 are mediated primarily by the activation and expansion of CTL and NK cells, which are capable of recognizing and eliminating tumor cells. However, this immunostimulatory effect is balanced by the expansion of regulatory T cells, which preferentially express CD25 and can reduce effector responses, highlighting the inherently paradoxical role of the cytokine [14].

In a study evaluating circulating interleukins as biomarkers in cutaneous melanoma, interleukin-2 (IL-2) showed significant prognostic potential. Plasma IL-2 levels were elevated in melanoma patients compared to healthy controls at early Breslow stages, suggesting its relevance in early detection of the disease. Notably, IL-2 levels were strongly correlated with sentinel lymph node status. Patients with positive sentinel lymph node metastases showed significantly higher median IL-2 concentrations than those with negative sentinel lymph nodes. This significant correlation suggests that increased systemic IL-2 levels reflect more advanced biological behaviors and metastatic potential. Together, these findings support a role for IL-2 as a prognostic biomarker in cutaneous melanoma, particularly in relation to regional lymph node involvement [4].

Soluble interleukin-2 receptor (sIL-2R), a circulating marker of IL-2-mediated immune activation, has been investigated as a prognostic indicator of malignant melanoma. In a cohort study including 172 melanoma patients and 60 healthy controls, serum concentrations of sIL-2R were found to be significantly elevated in melanoma patients compared with controls. Notably, the highest levels of sIL-2R were observed in patients who developed metastatic disease during follow-up, while no significant association was detected between sIL-2R levels and initial stage of disease. Multivariate analysis using logistic and Cox regression models showed that elevated serum sIL-2R was the only variable significantly associated with metastatic progression, independent of age, sex, stage, and disease-free interval. In contrast, serum IL-6 levels showed correlations with demographic factors but did not predict disease progression. These findings may indicate that increased circulating sIL-2R reflects biological processes associated with melanoma dissemination and support its role as an independent prognostic biomarker for metastatic progression in melanoma [15].

The prognostic significance of serum levels of soluble interleukin-2 receptor (sIL-2R) was assessed in patients with stage I-III cutaneous melanoma. Elevated sIL-2R concentrations at baseline and increased sIL-2R levels during follow-up were associated with tumor progression and reduced disease-free survival. Notably, sIL-2R levels correlated with established pathological features of melanoma, including tumor thickness, and demonstrated prognostic relevance independent of conventional clinical parameters. Multivariate analyses identified baseline and dynamic patterns of sIL-2R as independent predictors of disease-free survival, in addition to key histopathological factors. These findings suggest that circulating sIL-2R reflects biologically relevant immune activation associated with melanoma progression and support its potential utility as a prognostic biomarker [16].

The evidence supporting IL-2 and sIL-2R as prognostic biomarkers in cutaneous melanoma comes predominantly from observational cohort studies with moderate sample sizes and heterogeneous patient populations. Strengths of the available data include consistent associations between elevated IL-2 or sIL-2R levels and markers of disease progression in independent cohorts, as well as the use of multivariate analyses that adjust for established clinicopathological factors. However, several limitations reduce the level of evidence. Most studies were conducted before the widespread use of immune checkpoint inhibitors, limiting their applicability to contemporary treatment eras and current therapeutic decision-making. Differences in study design, disease stage distribution, study methodologies, and timing of cytokine measurements contribute to heterogeneity among studies and complicate direct comparison of results. Furthermore, the biology of IL-2 is inherently complex due to its dual role in promoting both effector and regulatory immune responses, which may partly explain the conflicting findings regarding its prognostic versus immunosuppressive effects. Although sIL-2R appears to be more reproducible as a marker of immune activation and risk of metastasis, prospective validation in larger, uniformly treated cohorts is lacking. Overall, IL-2 and sIL-2R currently represent biologically plausible but exploratory prognostic biomarkers, with insufficient evidence to support routine clinical use without further validation in the context of modern immunotherapy strategies.

## 3. Interleukin 6

Interleukin-6 (IL-6) is a pleiotropic cytokine with central roles in inflammation, immune regulation, and tumor biology [17]. In cutaneous melanoma, IL-6 has been widely implicated both as a mediator of tumor progression and as a circulating biomarker [18,19]. It is produced by melanoma cells and multiple stromal components, including fibroblasts, endothelial cells, macrophages, and lymphocytes, enabling autocrine and paracrine signaling that influences tumor growth, immune interactions, and systemic inflammation. [19,20,21,22,23,24,25]. Through these pathways, IL-6 links local tumor biology to systemic disease manifestations.

IL-6 signaling begins with binding to IL-6Rα and recruitment of the gp130 subunit, activating downstream pathways including JAK/STAT3, MAPK, and PI3K/Akt [26,27,28,29]. STAT3-driven transcription promotes proliferation, survival, angiogenesis, and apoptosis resistance, and immune suppression, and persistent STAT3 activation in melanoma is linked to increased metastasis and therapy resistance [30,31,32].

IL-6 supports melanoma survival under metabolic or oxidative stress and increases resistance to apoptosis, promoting tumor persistence in inflammatory conditions [26,33]. It also indirectly enhances angiogenesis by stimulating vascular growth factors and altering endothelial behavior, promoting neovascularization [34,35]. Beyond tumor cells, IL-6 influences immune cell recruitment and differentiation [30,36,37]. Thus, IL-6 links inflammation, tumor growth, and immune dysfunction.

Clinically, elevated serum IL-6 levels have been observed in patients with cutaneous melanoma, particularly in those with advanced or metastatic disease [38,39]. Compared with healthy individuals or patients with localized disease, metastatic melanoma patients exhibit markedly higher circulating IL-6 concentrations that correlate with tumor burden [4,39]. Importantly, these elevations appear largely independent of demographic variables, including age and sex, indicating that serum IL-6 predominantly reflects tumor-associated inflammation rather than patient-specific host characteristics. IL-6 has been shown to have significant prognostic value, reflecting underlying disease aggressiveness rather than treatment-specific effects. Elevated baseline serum IL-6 levels are associated with shorter progression-free survival and overall survival across multiple patient cohorts [39,40]. These associations remain significant after adjustment for established prognostic factors, including disease stage and lactate dehydrogenase levels, indicating that IL-6 provides additional biologically relevant insight into tumor aggressiveness [23].

IL-6 retains its prognostic value across multiple treatment modalities, including chemotherapy, cytokine-based immunotherapy, and immune checkpoint inhibition, suggesting that it reflects the underlying disease biology rather than just treatment-specific effects [18,38]. Longitudinal monitoring of IL-6 levels further highlights its clinical relevance. Persistently elevated or rising IL-6 during therapy correlates with disease progression and poor survival, whereas stable or declining levels are often observed in patients achieving disease control [38]. These fluctuations support the potential utility of IL-6 as a biomarker for real-time disease monitoring. These clinical associations may result from IL-6’s effects on the immune system, as elevated levels can promote immune evasion and reduce responses to immunotherapy by expanding regulatory and suppressive myeloid populations [19,26]. Study by Rossi et al. showed that patients with elevated IL-6 may derive less benefit from immune checkpoint inhibitors, suggesting a potential predictive association in this specific treatment context, underscoring the potential role of IL-6 as a stratification biomarker in personalized therapy [41]. Emerging evidence also suggests that baseline IL-6 levels may be associated with the risk of immune-related toxicity, rather than treatment efficacy, in patients receiving checkpoint inhibitors. In a prospective study of 140 metastatic melanoma patients treated with the CTLA-4 inhibitor ipilimumab, low baseline serum IL-6 levels were significantly and independently associated with a higher risk of severe immune-related adverse events, alongside female sex as an additional risk factor [42]. This finding indicates that circulating IL-6 may help identify patients at increased risk for immunotherapy-associated toxicity and could inform personalized surveillance strategies during treatment.

Overall, IL-6 serves as a biologically and clinically informative cytokine in cutaneous melanoma, with great prognostic significance and emerging, context dependent predictive associations that require prospective validation. Its roles in promoting tumor growth, angiogenesis, and immune suppression, along with its strong correlation with disease burden and survival outcomes, supports its inclusion in multiparametric biomarker panels for prognostic stratification and therapeutic monitoring. Future studies should aim to integrate IL-6 assessment into clinical practice and explore therapeutic strategies targeting IL-6 signaling to enhance patient outcomes. In particular, IL-6 receptor inhibition with tocilizumab has shown promise as a therapeutic option for managing immune-related adverse events associated with checkpoint inhibitor therapy, demonstrating clinical benefit and manageable safety profiles in patients with immune checkpoint inhibitor induced toxicities and enabling continued immunotherapy in many cases [43,44].

Despite strong biological plausibility and consistent associations between elevated IL-6 levels and advanced disease, tumor burden, and poor survival, several limitations of the existing literature should be considered. Most studies are retrospective and observational, limiting causal inference and placing the overall level of evidence at a correlative level. Cohort heterogeneity, small sample sizes, and variability in assay methods and cutoff values contribute to reproducibility concerns. Conflicting findings, particularly regarding the predictive value of IL-6 for response to immune checkpoint inhibitors, likely reflect differences in study design and patient populations. As IL-6 levels are influenced by systemic inflammation and comorbidities, it is not yet suitable as an independent clinical biomarker and is currently best positioned as part of integrated, multiparametric prognostic models pending confirmation in prospective studies.

## 4. Interleukin 8

Interleukin-8 (IL-8), also known as CXCL8, is a pro-inflammatory chemokine primarily recognized for its role in neutrophil recruitment [45,46]. In cutaneous melanoma, IL-8 drives tumor progression, angiogenesis, and immune modulation, and serves as a clinically relevant circulating biomarker. It signals via CXCR1 and CXCR2, receptors expressed on melanoma cells, endothelial cells, and multiple immune cell types in the tumor microenvironment, enabling IL-8 to impact tumor, stromal, and immune compartments [47,48].

IL-8 is continuously produced by melanoma cells, and its expression is further increased by oncogenic mutations, inflammatory signals, and tumor hypoxia [49,50,51]. Autocrine IL-8 signaling enhances proliferation, survival, migration, and invasion by activating pathways involved in cytoskeletal remodeling, extracellular matrix degradation, and metastatic transcriptional programs [52,53]. Collectively, these effects support local tumor growth and systemic spread.

In addition to its effects on tumor cells, IL-8 is a strong pro-angiogenic factor that promotes endothelial proliferation, migration, and survival, driving neovascularization that sustains melanoma growth [54,55,56]. By linking inflammatory signaling to angiogenesis, IL-8 creates a microenvironment that supports melanoma progression [57]. Consequently, IL-8 is a key mediator of proliferation, invasion, and vascularization. Clinically, circulating IL-8 levels strongly correlate with tumor burden. A study by Scheibenbogen et al. showed that patients with metastatic melanoma have significantly higher serum IL-8 concentrations than healthy controls, and that these levels correlate with overall tumor load rather than the location of metastases [58]. IL-8 serum levels decrease following effective tumor resection or systemic therapy, whereas levels rise with disease progression, supporting its role as a dynamic biomarker of tumor activity [52,59].

The prognostic value of IL-8, defined by its association with survival outcomes independent of therapy, has been consistently demonstrated. Elevated baseline IL-8 levels predict shorter progression-free and overall survival, independent of conventional prognostic factors [50,60]. This prognostic relevance is observed across treatment modalities, including targeted therapies and immune checkpoint inhibition, despite the ability of targeted therapies to remodel the tumor microenvironment and promote immune resistance, suggesting that IL-8 reflects fundamental biological characteristics of aggressive melanoma rather than purely therapy-specific effects [52,61].

In addition to its prognostic value, IL-8 utilizes significant effects on the tumor immune microenvironment. It recruits neutrophils and other myeloid populations with immunosuppressive functions, creating an inflammatory milieu that suppresses effective anti-tumor immunity [61,62]. Elevated IL-8 expression has been associated with increased intratumoral neutrophil infiltration and reduced benefit from immune checkpoint inhibitors, providing mechanistic insight into its association with adverse outcomes [63]. By altering immune cell composition and function, IL-8 promotes immune evasion and resistance to therapy.

Its potential as a longitudinal marker makes IL-8 especially valuable in monitoring melanoma progression. Longitudinal monitoring of IL-8 during systemic treatment indicates that early reductions correlate with tumor response, whereas persistently elevated or rising levels predict radiographic progression [50,64]. A study conducted by Sanmamed et al. showed that, in metastatic melanoma patients treated with anti-PD-1 antibodies (nivolumab or pembrolizumab), early decreases in serum IL-8 levels correlated with objective tumor responses. In contrast, stable or rising levels were associated with disease progression and could help distinguish true progression from immune-related pseudo progression, a major clinical challenge in melanoma management [65]. These findings suggest that monitoring IL-8 levels can complement imaging to guide treatment evaluation and clinical decision making.

Overall, IL-8 represents a cytokine of significant biological and clinical relevance in cutaneous melanoma. IL-8’s roles in tumor growth, invasion, angiogenesis, and immune regulation, together with its correlation with tumor burden and dynamic treatment responses, make it a key element in multiparametric cytokine profiling. Incorporating IL-8 into clinical monitoring could enhance personalized prognostic evaluation and support the optimized management of patients with advanced melanoma.

While strong mechanistic and clinical data support the role of IL-8 in melanoma progression, the current evidence has limitations. Most studies are observational, providing primarily correlative evidence. Study heterogeneity with respect to disease stage, treatment exposure, timing of sampling, and assay platforms contributes to variability in reported thresholds and effect sizes. While many studies support a predictive role for IL-8 in immunotherapy response, conflicting findings exist, reflecting differences in study design and patient selection. Additionally, IL-8 levels can be influenced by systemic inflammation and non-tumor factors, causing reproducibility concerns. Consequently, IL-8 is not yet validated for clinical use as a standalone biomarker and should be interpreted mainly within multiparametric panels until confirmed in prospective standardized trials.

## 5. Interleukin 10

Chronic inflammation is recognized as a fundamental factor contributing to carcinogenesis, facilitating molecular and cellular events that promote malignant transformation and tumor progression. Cytokines are central mediators of these processes, as they regulate immune responses, cell proliferation, and survival within the tumor microenvironment. Among these mediators, interleukin-10 (IL-10), a potent anti-inflammatory cytokine, has been extensively studied for its dual immunomodulatory functions, both tumor growth-promoting and tumor-inhibiting properties [66]. In some experimental systems, IL-10 has been reported to have antiangiogenic effects, while, in others, particularly in melanoma, its immunosuppressive effects appear to support tumor progression [67].

IL-10 is a key immunoregulatory cytokine with a central role in maintaining immune homeostasis and limiting excessive inflammatory responses [68]. It is produced by a wide range of immune cells, including regulatory T cells, type 1 regulatory T cells, macrophages, dendritic cells, and B cells, as well as certain tumor cells [69,70]. IL-10 exerts its biological effects by binding to the IL-10 receptor heterotetrameric complex, which leads to the activation of the JAK1-STAT3 signaling pathway. This signaling cascade results in transcriptional programs that suppress the production of proinflammatory cytokines, inhibit antigen presentation, and promote immune tolerance [71,72]. In the context of cancer, IL-10 is strongly associated with shaping the tumor microenvironment by dampening antitumor immune responses and facilitating immune escape mechanisms [73].

IL-10 contributes to immune evasion in melanoma through multiple pathways. By reducing the expression of HLA class I and II molecules and intercellular adhesion molecule-1 (ICAM-1) on melanoma cells, IL-10 reduces their visibility to cytotoxic T lymphocytes and interferes with effective immune recognition. This reduced expression also impairs dendritic cell maturation and APC function, leading to reduced T-cell generation and reduced antitumor immune surveillance. IL-10 has been shown to act as an autocrine growth factor in melanoma cells: melanoma cell cultures exposed to IL-10 showed enhanced proliferation and prolonged survival, while inhibition of IL-10 reduced cell proliferation. The expression of the IL-10 receptor (IL-10R) on melanoma cells supports these direct effects on tumor cell biology [74].

Some studies have shown that IL-10 is actively expressed within melanoma tissue and contributes to the immunological landscape of the tumor microenvironment [75,76,77]. Analyses of melanoma samples and cell populations have shown the presence of IL-10 at both the transcript and protein levels, whereas its expression is minimal or absent in non-malignant melanocytic cells. Importantly, IL-10 within melanoma lesions originates from multiple cellular sources, including malignant melanoma cells as well as tumor-infiltrating immune populations such as macrophages and lymphocytes. This diverse cellular contribution supports the existence of both autocrine and paracrine IL-10 signaling pathways, which together shape local immunosuppression and influence tumor–immune interactions [76].

In cutaneous melanoma, IL-10 has emerged as a key mediator of tumor-immune interactions. Increasing evidence indicates that the expression of IL-10 in melanoma cells is closely related to tumor progression and the acquisition of metastatic potential [4]. Analysis of primary melanomas at different evolutionary stages has shown a significant increase in IL-10 expression during the transition from radial to vertical growth phase, a key step in melanoma aggressiveness. IL-10 mRNA is rarely detected in melanoma in situ but becomes predominant in invasive and metastatic lesions, suggesting a stage-dependent upregulation. Importantly, higher IL-10 expression correlates with established histopathological markers of poor prognosis, including Clark’s score and vertical growth phase dominance. In addition to tumor cells, IL-10 production by melanoma-associated macrophages and lymphocytes further contributes to local immunosuppression. This cytokine-mediated immunomodulation extends to sentinel lymph nodes, where tumor-derived IL-10 may impair immune surveillance and facilitate early metastatic spread. Together, these findings support a central role for IL-10 in remodeling the tumor microenvironment, promoting immune escape and promoting melanoma progression, thereby highlighting its potential importance as a biomarker for disease stratification [78].

The study by Rendleman et al. highlighted the prognostic relevance of immunomodulatory gene variants in cutaneous melanoma, underscoring the need for biomarkers beyond conventional clinicopathological parameters. By analyzing germline polymorphisms in genes involved in immune regulation, the authors identified significant associations between overall survival and genetic variants located at the 1q32.1 locus, which encompasses several interleukin genes. It is particularly important to note that a regulatory single-nucleotide polymorphism within the IL10 gene showed the strongest association with survival outcomes. These findings were further supported by transcriptomic data from metastatic melanoma samples, where IL10 expression levels correlated with overall survival. Together, this study provides robust evidence that both genetic variation and IL-10 expression influence melanoma prognosis, highlighting the clinical potential of IL-10-related markers for personalized prognostic stratification [79].

Serum IL-10 levels have been investigated as indicators of systemic immune regulation. Some clinical studies comparing melanoma patients with healthy controls have found higher circulating IL-10 in melanoma patients [80,81]. It is important to note that elevated serum IL-10 levels are associated with poorer survival outcomes. In one prospective comparison, melanoma patients with IL-10 levels above a defined threshold showed a significantly shorter median survival compared with those with lower IL-10 levels. This observation supports the concept that systemic IL-10 may reflect an immunosuppressive state associated with more aggressive disease [80].

A study by Mahipal et al. investigated the relationship between IL-10 production by melanoma cells and clinical outcome in patients with advanced cutaneous melanoma treated with an autologous, hapten-modified melanoma vaccine. Melanoma cells isolated from metastatic lesions were evaluated in vitro for their ability to produce IL-10 prior to vaccine preparation. Patients were then stratified based on IL-10 production levels, and survival outcomes were analyzed using established statistical models. The findings suggest that intrinsic IL-10 production by tumor cells reflects biologically relevant differences within the tumor microenvironment that may influence therapeutic response, particularly in the adjuvant setting. Overall, the study supports the concept that tumor-derived IL-10 contributes to melanoma immunomodulation and may represent a factor of prognostic importance in patients receiving immunotherapeutic interventions [82].

The role of IL-10 in cutaneous melanoma is supported by a diverse body of evidence, including tissue-based expression analyses, serum biomarker studies, genetic association data, and selected treatment cohorts. A key strength of the literature is its biological consistency at multiple levels, with concordant findings at the transcriptomic, protein, and germline gene levels linking IL-10 to tumor progression and adverse outcomes. However, the overall level of evidence remains limited by significant heterogeneity in study design, patient cohorts, and disease stages, ranging from early primary melanomas to advanced metastatic disease. Many studies are cross-sectional or retrospective and rely on relatively small sample sizes, which limits causal inference and reproducibility. In addition, IL-10 expression originates from multiple cellular sources within the tumor microenvironment, complicating the interpretation of whether measured IL-10 reflects intrinsic tumor aggressiveness, host immunosuppression, or both. The conflicting results regarding circulating IL-10 levels may also be influenced by differences in assay sensitivity, sampling time, and systemic inflammatory comorbidities. It is important to note that most studies were conducted before the routine use of immune checkpoint inhibitors, and the relevance of IL-10 as a prognostic or predictive biomarker in the modern era of immunotherapy remains unclear. Although IL-10 represents a biologically plausible mediator of immune escape and disease progression, its current clinical readiness is limited, and prospective validation in well-defined, uniformly treated cohorts is needed before routine clinical application.

## 6. Interleukin 17

Interleukin-17 (IL-17) is a proinflammatory cytokine that has emerged as a mediator at the border between immunity, inflammation and cancer [83]. In the context of cutaneous melanoma, IL-17 plays a complex and often paradoxical role, contributing to both tumor-promoting and antitumor immune responses, depending on the cellular source, tumor microenvironment, and stage of the disease [84].

IL-17 primarily refers to IL-17A, the founding member of the IL-17 cytokine family, which also includes IL-17F, IL-17C, and others [5]. IL-17A is predominantly produced by a distinct subset of CD4^+^ T helper cells known as Th17 cells, but can also be secreted by γδ T cells, natural killer (NK) cells, innate lymphoid cells, and neutrophils [85,86]. Differentiation and maintenance of Th17 cells are regulated by cytokines such as IL-6, IL-1β, TGF-β and IL-23, all of which are often dysregulated in melanoma. As a result, elevated levels of IL-17 are frequently detected in the melanoma tumor microenvironment (TME), as well as in the peripheral blood of patients with advanced disease [84,87].

IL-17 exerts its effects by binding to the IL-17 receptor complex, which leads to the activation of downstream signaling pathways, including NF-κB, MAPK, and STAT3. These pathways regulate the expression of pro-inflammatory mediators such as IL-6, TNF-α, IL-8 and granulocyte colony-stimulating factor (G-CSF) [83,88,89]. This inflammatory signaling cascade can promote tumor growth by enhancing angiogenesis, recruiting myeloid-derived suppressor cells (MDSCs), and promoting an immunosuppressive microenvironment [90,91].

In cutaneous melanoma, characterization of IL-17-producing tumor-infiltrating lymphocytes revealed a relatively low prevalence of Th17 cells compared with basal cell carcinoma, a nonmelanoma skin cancer enriched in this subset. Both immunohistochemical and ex vivo analyses showed reduced Th17 infiltration in melanoma lesions, and melanoma-derived TILs could not be induced to differentiate towards a Th17 phenotype in vitro. The presence of Th17 cells did not correlate with clinical parameters, although a lower frequency was observed in samples from female patients. The trend toward association with placental growth factor expression in melanoma cells suggests a potential link between angiogenic signaling and local Th17 responses, highlighting the need for further investigation before assigning prognostic or therapeutic relevance to IL-17 in melanoma [92].

IL-17 has attracted interest as part of a cytokine network under investigation for its potential relevance in the pathology of cutaneous melanoma. Immunohistochemical analysis of melanoma samples showed significantly higher expression levels of IL-17 and the Th17-associated cytokine IL-23 compared to benign melanocytic and Spitz nevi. Increased expression of IL-17 was observed both in staining intensity and in the proportion of positive cells, although it did not correlate with established prognostic parameters such as Breslow thickness or Clark level. Concomitant overexpression of p73, which shows characteristic cytoplasmic localization, suggests the potential involvement of the IL-17/IL-23 axis in melanoma biology, possibly contributing to tumor invasiveness and not just tumor burden [93].

IL-17 has emerged as a context-dependent mediator of immune responses in cutaneous melanoma, with limited evidence supporting a general prognostic role. Recent studies showed that tumors carrying BRAFV600 mutations show an enrichment of Th17 gene expression signatures associated with IL-17, which are associated with increased infiltration of T-cells and neutrophils. High baseline IL-17 signature values consistently predicted clinical responses to combined immune checkpoint inhibition of anti-PD-1 and anti-CTLA-4, whereas no such association was observed with monotherapy. Functional analyses revealed that both IL-17A inhibition and neutrophil depletion reduced the efficacy of dual immune checkpoint inhibition and reduced T-cell activation. Elevated circulating IL-17A levels also reflected a systemic Th17 cytokine profile that preceded clinical response, highlighting the potential of IL-17 as a predictive biomarker for patient stratification in dual immune checkpoint inhibition therapy [94].

The evidence about IL-17 in cutaneous melanoma is characterized by marked heterogeneity and context-dependent findings, reflecting differences in tumor biology, molecular subtypes, and treatment settings. Early tissue-based studies suggested a limited presence of Th17 cells in melanoma lesions and a lack of consistent association with clinicopathological parameters, suggesting poor prognostic relevance in unselected patient populations. In contrast, more recent molecular and functional studies of BRAFV600-mutated melanoma provide stronger, mechanistic evidence linking IL-17-related immune signatures to treatment response, particularly in the context of combined immune checkpoint inhibition. The strengths of these studies include integrated transcriptomic analyses, functional validation, and correlation with clinical outcomes. However, limitations remain significant. Many of the findings were derived from relatively small, highly selected cohorts and may not be generalizable to other melanoma subtypes or treatment modalities. Differences between immunohistochemical, circulating, and gene-expression-based measurements further complicate interpretation and reproducibility. It is important to note that the predictive value of IL-17 appears to be treatment and mutation specific (e.g., BRAFV600-mutated disease treated with combined checkpoint inhibition) rather than broadly applicable, and that most of the supporting data come from the modern era of immunotherapy, limiting comparison with earlier studies. Overall, IL-17 currently represents a promising but highly contextual predictive biomarker rather than a robust prognostic factor, and its clinical readiness depends on further prospective validation in molecularly stratified cohorts receiving standardized immunotherapy regimens.

## 7. Interleukin 18

Interleukin-18 (IL-18) is a pro-inflammatory cytokine belonging to the IL-1 superfamily, initially discovered as an interferon-γ (IFN-γ)–inducing factor [95,96]. IL-18 is produced as an inactive precursor and requires caspase-1–mediated cleavage to produce its biologically active form [3,97]. A broad range of cell types, including monocytes, macrophages, dendritic cells, endothelial cells, and lymphocytes, can produce IL-18, allowing it to participate in both innate and adaptive immune responses [3,98]. In cutaneous melanoma, IL-18 has complex, context-dependent effects, shaping tumor–host interactions, modulating immune surveillance, and impacting clinical outcomes.

IL-18 exerts its biological activity via a heterodimeric receptor complex composed of IL-18Rα and IL-18Rβ subunits, which activate NF-κB and MAPK signaling pathways [99]. A key immunological function of IL-18 is the induction of IFN-γ production by natural killer (NK) cells, particularly in the presence of costimulatory signals, thereby enhancing cytotoxic immune responses critical for tumor immunosurveillance [100,101]. This ability to promote anti-tumor immunity distinguishes IL-18 from other inflammatory cytokines that primarily facilitate tumor-promoting inflammation.

Experimental studies in melanoma models indicate that IL-18 can also modulate tumor cell behaviors directly. A study by Hue et al. showed that, in murine melanoma cells, IL-18 stimulates the expression of growth-promoting factors such as stem cell factor (SCF), suggesting that IL-18 may support tumor cell survival and proliferation under certain conditions [102]. A study by Vidal-Vanaclocha et al. demonstrated that, in preclinical models of hepatic metastasis, tumor-derived IL-18 increased melanoma cell adhesion to hepatic sinusoidal endothelial cells by upregulating vascular cell adhesion molecule-1 (VCAM-1), thereby facilitating metastatic colonization [103].

Despite these tumor-promoting effects being seen in experimental models, analyses of human melanoma datasets reveal an opposing clinical association. Higher intratumoral IL-18 expression correlates with improved overall survival and increased infiltration of cytotoxic immune populations, including CD8^+^ T cells and NK cells, as well as elevated expression of cytolytic molecules such as perforin and granzyme B [104,105]. These findings indicate that, in the human disease context, IL-18 may reflect an active anti-tumor immune microenvironment that promotes immune-mediated tumor control.

IL-18’s capacity to stimulate antitumor immunity has prompted studies exploring its therapeutic applications. Early clinical studies evaluating recombinant IL-18 in advanced melanoma showed acceptable safety but limited objective responses when used as a monotherapy [105]. Evidence from preclinical models suggests that IL-18 may synergize with costimulatory signals or activated immune effector cells to enhance antitumor immunity, highlighting its potential in combination therapies rather than as monotherapy [105].

Collectively, IL-18 plays a multifaceted role in cutaneous melanoma. While experimental studies underscore IL-18’s potential to enhance tumor proliferation and metastasis in specific contexts, analyses of human melanoma datasets associate elevated IL-18 levels with heightened immune activity and favorable patient outcomes [103,104,105]. This duality highlights the context-dependent nature of IL-18 signaling and the critical role of the tumor microenvironment in determining its biological effects. Overall, IL-18 holds promise both as a biomarker of immune-mediated tumor control and as a potential target in combination immunotherapy strategies to enhance antitumor immunity.

Although IL-18 has been implicated in melanoma biology, the available evidence is heterogeneous and remains largely exploratory. Clinical associations are mainly derived from retrospective cohorts and transcriptomic datasets, with limited sample sizes and variable methodologies, including differences in sample type (serum versus tumor tissue), assay platforms, and adjustment for confounders such as stage, treatment history, and immune infiltration. These factors contribute to inconsistencies across studies and raise reproducibility concerns. Moreover, preclinical models demonstrate that IL-18 can exert tumor promoting effects under specific conditions, highlighting the context dependent nature of its signaling. Consequently, the overall level of evidence remains limited, and IL-18 is not clinically ready as to be used as a standalone biomarker. It may be more appropriately evaluated as part of multiparametric biomarker panels, pending validation in prospective, standardized studies.

## 8. Clinical Implications and Integration into Personalized Melanoma Management

Although interleukins have traditionally been studied as individual immune mediators, increasing evidence suggests that their greatest clinical value lies in their integration with established prognostic and predictive frameworks for melanoma. Table 1 outlines the integration of interleukin signatures with established clinical and molecular parameters, illustrating their potential application in personalized melanoma management. Current risk stratification relies primarily on clinicopathological parameters such as AJCC stage, serum lactate dehydrogenase (LDH) levels, and molecular features, including BRAF and NRAS mutational status. However, these parameters do not fully capture the dynamic immunological landscape that influences disease progression and therapeutic response. Cytokine profiling, particularly circulating interleukin signatures, offers an additional layer of biological information that reflects tumor and host immune system interactions. Elevated levels of IL-6 and IL-8 are consistently associated with high tumor burden, advanced disease stage, elevated LDH, and poor survival outcomes. Integrating these cytokines into existing prognostic models may improve differentiation within AJCC stages III-IV disease, identifying biologically aggressive tumors that could benefit from early treatment or closer surveillance.

Interleukins can influence therapeutic decision making in the era of immune checkpoint inhibitors. Proinflammatory and immunosuppressive cytokines have opposing effects on antitumor immunity, and their balance may influence treatment efficacy. IL-17-related immune signatures have been associated with favorable responses to combined anti-PD-1 and anti-CTLA-4 therapy, particularly in melanoma with a BRAFV600 mutation, suggesting potential utility in treatment selection. On the other hand, elevated baseline IL-6 and IL-8 levels are associated with primary resistance to immune checkpoint inhibitors and poorer outcomes, supporting their role as negative predictive biomarkers.

In addition to initial risk assessment, dynamic monitoring of interleukin levels during therapy may provide clinically applicable insights. Changes in IL-6, IL-8, and IL-10 during treatment can help distinguish pseudo progression from true disease progression, a major challenge in melanoma patients treated with immunotherapy. Rising cytokine levels in the absence of radiological progression might signal the emergence of resistance or immune escape mechanisms, potentially prompting earlier treatment adaptation. Finally, these observations support a model in which interleukin profiling complements established prognostic markers, allowing for more precise risk stratification and more personalized therapeutic strategies. Prospective clinical trials incorporating cytokine-guided algorithms are needed to confirm their utility in routine melanoma management. Table 2 summarizes the key biological functions and clinical associations of selected interleukins in cutaneous melanoma, highlighting their prognostic and predictive relevance.

## 9. Integrated Cytokine Signatures and Multi-Parametric Biomarker Models

Contemporary oncology increasingly recognizes that single biomarkers rarely capture the complexity of tumor biology or treatment response. In melanoma, immune regulation is orchestrated through joined cytokine networks rather than isolated mediators, highlighting the need for multivariate biomarker approaches. Integrated cytokine signatures, incorporating multiple interleukins with complementary biological functions, might therefore provide greater prognostic and predictive performance compared with single-cytokine analyses.

Multi-parametric models combining IL-6, IL-8, IL-10, IL-17, and IL-18 reflect distinct immune phenotypes, ranging from immunosuppressive, tumor-promoting inflammation to effective antitumor immune activation. For example, a profile characterized by elevated IL-6 and IL-8 alongside increased IL-10 could identify patients with systemic inflammation and immune exhaustion, associated with poor outcomes and limited immunotherapy responsiveness. In contrast, signatures high in IL-17 and IL-18 could indicate preserved immune competence and favorable response to immune checkpoint inhibition.

Advances in multiplex immunoassays, machine learning, and systems biology approaches further facilitate the development of cytokine-based predictive algorithms. These tools may support individualized treatment selection, early detection of therapeutic resistance, and adaptive therapeutic strategies. Future prospective studies and clinical trials incorporating integrated cytokine panels are essential to establish standardized thresholds, validate clinical utility, and translate these models into routine practice.

## 10. Conlusions

Cytokine profiling represents a powerful framework for capturing the complexity of immune regulation in cutaneous melanoma. Interleukins, as central modulators of immune signaling, mirror the dynamic balance between tumor progression and host immune surveillance. Specifically, IL-2 emerges as a marker of early disease biology, with elevated levels correlating with sentinel lymph node positivity and underscoring its prognostic relevance. In contrast, IL-6 and IL-8 consistently track with tumor burden, advanced disease stage, and inferior survival outcomes, highlighting their value as indicators of aggressive tumor behavior. IL-10 expression mirrors tumor progression and immune suppression, reinforcing its role in shaping an immunomodulatory tumor microenvironment. IL-17 signatures demonstrate predictive utility, particularly for response to combined immune checkpoint inhibition in BRAFV600-mutant melanoma, while IL-18 exhibits context-dependent duality, associating with both immune activation and favorable outcomes depending on tumor biology. Collectively, their pleiotropic and context-dependent effects highlight the need for integrated cytokine signatures rather than reliance on single biomarkers. As analytical technologies continue to advance, interleukin-based profiling offers great potential to enhance prognostic stratification and improve clinical risk assessment. Importantly, the integration of cytokine profiles into translational research supports the paradigm shift toward personalized medicine, enabling more precise patient stratification and the development of individualized therapeutic strategies in cutaneous melanoma. Future studies should focus on prospective validation in large, uniformly treated melanoma cohorts within the modern immunotherapy era. Standardized assay methods, predefined cutoff values, and consistent sampling time points will be crucial to improve reproducibility and cross-study comparability. Given the context-dependent and pleiotropic effects of cytokine signaling, these markers should be evaluated within multiparametric models integrating circulating, tissue-based, and molecular data rather than as standalone biomarkers. Longitudinal analyses are particularly important to help distinguish baseline prognostic value from dynamic predictive effects during treatment. Only rigorously designed prospective studies with standardized methodologies and clinically meaningful outcome measures can determine whether these cytokines can be translated into clinically applicable tools for evidence-based management of cutaneous melanoma.

## Figures and Tables

**Table 1:** Translational integration of interleukin signatures into personalized melanoma management.

Clinical Setting	Interleukin Markers	Integrated with	Potential Clinical Utility	Supporting References
Baseline prognostic stratification	IL-6, IL-8, IL-10	AJCC stage, LDH	Improved risk discrimination in stage III–IV melanoma	[4,15,18,38,39,58]
Prediction of immunotherapy response	IL-6, IL-8, IL-17, IL-18	BRAF status, treatment type	Identification of patients more likely to benefit from immune checkpoint inhibition	[7,41,53,63,94,104]
Monitoring during treatment	Dynamic IL-6, IL-8, IL-10 changes	Radiological and clinical data	Early detection of emerging resistance; differentiation between pseudo progression and true progression	[50,59,65]
Immune toxicity and treatment modulation	IL-6	Clinical toxicity grading	Prediction and management of immune-related adverse events	[42,43,44]
Multi-parametric biomarker models	IL-6, IL-8, IL-10, IL-17, IL-18 panels	Molecular and immune biomarkers	Development of integrated immune signatures for precision oncology	[1,2,7,40,64]

Summary of potential clinical applications of interleukin-based biomarkers in melanoma. Most interleukins listed are not yet validated for routine clinical use and should be interpreted within multiparametric models together with established clinical and molecular markers.

**Table 2:** Biological and clinical significance of selected interleukins in cutaneous melanoma.

Interleukin	Key Biological Functions	Evidence in Melanoma	Clinical Relevance	Key References
IL-2	T-cell proliferation, activation of cytotoxic T lymphocytes, immune stimulation	Altered circulating IL-2 and soluble IL-2 receptor levels correlate with disease stage and immune activation	Prognostic relevance in early disease; historical and ongoing therapeutic relevance in melanoma immunotherapy	[4,8,9,10,11,12,13,14,15,16]
IL-6	Pro-inflammatory signaling, STAT3 activation, tumor growth, angiogenesis, immune suppression	Elevated serum IL-6 associated with tumor burden, advanced stage, poor survival and immunotherapy outcomes	Negative prognostic biomarker; predictive of response and toxicity to immune checkpoint inhibitors	[15,18,19,20,21,22,23,24,38,39,40,41,42,43]
IL-8 (CXCL8)	Angiogenesis, neutrophil recruitment, tumor invasion, metastatic dissemination	Increased serum and tumor IL-8 correlate with metastatic disease, tumor load and poor response to immunotherapy	Strong negative prognostic and predictive biomarker; reflects tumor burden and treatment response	[49,50,51,52,53,58,59,60,61,62,63,64,65]
IL-10	Immunosuppression, inhibition of antigen presentation, promotion of immune tolerance	Tumor-derived and circulating IL-10 associated with melanoma progression and metastatic competence	Context-dependent prognostic biomarker; marker of immune escape mechanisms	[66,67,68,69,70,71,72,73,74,75,76,77,78,79,80,81,82]
IL-17	Th17-mediated inflammation, immune modulation, cytokine network amplification	IL-17 signaling linked to immune activation and response to combined checkpoint inhibition	Predictive biomarker for benefit from dual CTLA-4/PD-1 inhibition	[83,84,85,86,87,88,89,90,91,92,93,94]
IL-18	IFN-γ induction, activation of NK and CD8^+^ T cells, immune regulation	Dual role in melanoma: immune activation versus tumor-promoting effects depending on context	Prognostic biomarker linked to immune infiltration and outcome	[95,96,97,98,99,100,101,102,103,104,105]

Overview of key biological functions, supporting evidence, and potential clinical relevance of selected interleukins. Most markers are still exploratory and not validated for routine clinical implementation; their interpretation should be integrated into multiparametric prognostic and predictive models.

## Data Availability

No new data were created or analyzed in this review. Data sharing is not applicable to this article.
